# Strategies for cost-effectiveness analysis of rehabilitation for older patients with acute heart failure

**DOI:** 10.1186/s12962-022-00387-8

**Published:** 2022-09-25

**Authors:** Kotomi Sakai, Ryo Momosaki, Eri Hoshino

**Affiliations:** 1 Comprehensive Unit for Health Economic Evidence Review and Decision Support (CHEERS), Research Organization of Science and Technology, Ritsumeikan University, #209, Research Park Bid. No. 2, 134, Minami-machi, Chudoji, Simogyo-ku, 600-8813 Kyoto, Japan; 2 Department of Rehabilitation Medicine, Mie University Graduate School of Medicine, Mie, Japan

**Keywords:** Rehabilitation, Older adults, Cost-effectiveness analysis

## Abstract

The prevalence of heart failure (HF) is increasing in the ageing world population, and its burden on the medical and health economic fields is enormous. Rehabilitation is an essential component of the nonpharmacological treatment of patients with HF; however, its efficacy and cost-effectiveness for patients with acute HF remain unclear. A trial assessed the cost-effectiveness of acute cardiac rehabilitation among older adults. Herein, we discussed strategies for the cost-effectiveness analysis of acute cardiac rehabilitation using the rehabilitation therapy in older acute heart failure patients trial.

## Background

Heart failure (HF) is a major public health issue, and its global burden on the medical and health economic sectors is enormous. The annual healthcare budget for HF is high, accounting for 1–2% of the total annual healthcare budget in Europe and the United States (US). In addition, the total health expenditure for HF in the US is expected to increase by 127% between 2012 and 2030 [1]. The incidence rate of HF has decreased in the last decades; however, its prevalence is increasing due to advancements in HF treatment methods and longer life-expectancy of HF patients [2]. Thus, it is important to focus on people living with HF. The key population in HF patients is older adults, in whom the most frequent cause of hospitalization is HF. In addition, 80% of hospitalizations and 90% of deaths in HF patients occur among older adults [3]. Due to the increase in the global ageing population and the increasing prevalence of HF, the burden of HF is expected to increase.

Exercise-based rehabilitation is a nonpharmacological method of HF treatment. A clinical guideline has reported that cardiac rehabilitation is useful for clinically stable patients with HF [4]. In contrast, due to the global lack of evidence of the benefits of cardiac rehabilitation in acute HF patients, rehabilitation in them is not common. In reality, the public health insurance policies of Medicare and Medicaid do not cover rehabilitation services for acute HF patients.

One important clinical trial involving older acute HF patients was conducted in the U.S., and subsequently the cost-effectiveness analysis (CEA) was published [5]. CEA is an important aspect for policy makers who are working with public health insurance systems. The study found that when rehabilitation intervention was compared with no-rehabilitation, the Incremental Cost-Effectiveness Ratio (ICER) of the rehabilitation was $58 409 per Quality Adjusted Life years (QALY). In the probabilistic sensitivity analysis, 45% and 54% of simulated ICERs were at or below the conventionally accepted thresholds of $50,000 and $100,000, respectively in the overall study participants including those with HF with preserved ejection fraction (HFpEF) and reduced ejection fraction (HFrEF). In contrast, improvements in outcomes were greater, and the cost-effectiveness was more in patients with HFpEF than in those with HFrEF. We would like to discuss four points based on the CEA using the Rehabilitation Therapy in Older Acute Heart Failure Patients (REHAB-HF) trial. The points discussed arose based on the Consolidated Health Economic Evaluation Reporting Standards 2022 (CHEERS 2022) checklist and were summarized in Table 1 [6].

**Table 1:** Summary of our comments on the CEA of acute cardiac rehabilitation and the corresponding items on the Consolidated Health Economic Evaluation Reporting Standards checklist 2022

	Item	Considerations for the CEA
Study population	5	Including patients with high demand
Comparators	7	Choosing best or common practice
Perspective	8	Choosing appropriate perspectives
Time horizon	9	Use of a relevant time-horizon
Rationale and description of model	16	Developing a model using relevant data

## Study population

The first point is the patient population in the trial. The REHAB-HF trial included approximately 55% of frail older adults, but excluded approximately 98% of patients with acute HF due to the following reasons: dementia, dependent on activities of daily living, planned discharge to a nursing home, and difficulties complying with trial requirements [7]. This could have contributed to the lack of generalizability of the results of the trial and led to the inaccurate evaluation of the cost-effectiveness of rehabilitation for acute HF patients. One of the evidence issues of randomized controlled trials on HF is that most of them exclude the oldest patients, whose need for treatment and care is increasing [8]. The mean age ± standard deviation was 72.7 ± 8.1 years in the REHAB-HF trial, and this implies that the oldest patients were excluded for reasons such as being dependent and having dementia. In CEAs for public health insurance systems, the proportion of patients with a particular characteristic such as the disease type and age is needed to be considered. In addition, the food and drug administration has issued guidance to enhance diversity in clinical trials and recommends including people with disability [9]. In future CEAs for acute cardiac rehabilitation, those who were excluded in the REHAB-HF trial should be considered.

## Comparators

The second point is the comparator in the trial. According to the 2022 AHA/ACC/HFSA Guideline for the Management of Heart Failure, it is recommended that such patients in the REHAB-HF trial should receive care from multidisciplinary teams as a nonpharmacological intervention [10]. To ensure external validity, a comparator should represent best or common practice [11]. Thus, the comparator should be care from multidisciplinary teams in the future CEAs for acute cardiac rehabilitation.

## Perspective

The third point is the cost related to caregiver burden. Both the patients and their caregivers should be considered in CEAs of trials including older adults who need some assistance in their activities of daily life. The Second Panel on Cost-effectiveness in Health and Medicine recommends using not only payer’s perspective, but also societal perspective, which considers the effect of the intervention on the patients, caregivers, and social resources in a CEA with a long-time horizon [12]. Caregiver burden in HF is a significant issue and can influence the health outcomes of both the patients and their caregivers [13]. Including the indirect costs such as the health status of caregivers and their lost opportunity and lost productivity costs in a CEA may be useful during an appraisal by policy makers.

## Modelling

Finally, there are two issues in the modelling for CEA. The time horizon used in the CEA of the REHAB-HF trial was lifetime. The time horizon must be long enough that the intended and unintended benefits and harms are captured [14]. In contrast, using a time horizon that is longer than necessary could add unnecessary cost, and interpreting the cost-effectiveness might become too difficult [14]. According to the CHEERS 2022 statement, giving reasons for setting a time horizon for a certain period is one of the domains [6]. In general, it is unlikely that the effects of rehabilitation in the acute phase of HF last throughout life, although the author of the article also mentioned that there is uncertainty in the duration of the effect of intervention [5]. In that case, using the available evidence from observational studies and expert opinions or referring to trials examining the duration of the effect of a similar intervention may be useful in setting the time horizon in a CEA. Moreover, conducting scenario analyses using some time horizons might help policy makers in making their decision. The second point is that the lifetime cost and QALY were estimated using a model that was developed and validated by trials involving patients with chronic HF who did not undergo acute cardiac rehabilitation [15]. Thus, modelling using the data of patients who underwent acute cardiac rehabilitation, e.g., data from a clinical trial with long-follow up or real-world data, can lead to a more accurate estimation of the cost-effectiveness.

## Conclusion

We discussed the strategy for the CEA of acute cardiac rehabilitation in older adults with HF using the REHAB-HF trial. Considering more frail older people (who can be a key population in HF in the ageing world) and the cost related to caregiver burden, and modelling with an appropriate time horizon and more accurate data may be useful in the future analysis of the cost-effectiveness of acute cardiac rehabilitation in older adults.

## Data Availability

Not applicable.

## Abbreviations

CEA: Cost-effectiveness analysis
CHEERS: Consolidated Health Economic Evaluation Reporting Standards
HF: Heart failure
ICER: Incremental cost-effectiveness ratio
QALY: Quality Adjusted Life years
REHAB-HF: Rehabilitation Therapy in Older Acute Heart Failure Patients
